# Is it feasible to nest a Trial within a Cohort Study (TwiCS) to evaluate an early years parenting programme? A Born in Bradford’s Better Start study protocol

**DOI:** 10.1186/s40814-023-01441-9

**Published:** 2024-01-30

**Authors:** Kate E. Mooney, Charlie Welch, Kirsty Crossley, Tracey Bywater, John Wright, Josie Dickerson, Sarah Blower

**Affiliations:** 1 Department of Health Sciences, University of York, York, UK; 2 Bradford Institute for Health Research, Bradford, UK

**Keywords:** Feasibility, Trial within a cohort study, Parenting programme, Birth cohort, Incredible years, Better Start Bradford, Born in Bradford’s Better Start

## Abstract

**Background:**

Evaluating the effectiveness of early years parenting interventions provides evidence to improve the development and wellbeing of children. This protocol paper describes a study to explore the feasibility of evaluating the Incredible Years Toddler early life intervention programme, which is offered to parents of 1–3-year-olds via the Better Start Bradford programme.

The study aims to use a Trial within a Cohort Study (TwiCS) design that randomly selects individuals participating in a cohort to be offered an intervention. The TwiCS information and consent process is person-centred and aims to replicate real-world practice whereby only those who are offered the intervention are given information about the intervention.

The cohort is the Born in Bradford’s Better Start (BiBBS) cohort, an interventional birth cohort recruiting expectant parents in three areas of Bradford, UK. The study will assess the feasibility of TwiCS procedures, staged consent, and intervention take-up.

**Methods:**

We will conduct a feasibility TwiCS to test study procedures. We aim to establish the following: (1) whether TwiCS methodology can be implemented to create control and intervention arms, whilst documenting any incidences of contamination within the cohort; (2) whether satisfactory rates of intervention uptake are achieved among participants allocated to the intervention; and (3) whether satisfactory rates of retention of participants in the intervention can be achieved. A Red Amber Green (RAG) rating system has been applied to support the feasibility assessment of each objective: to be rated red (not achieved), amber (partly achieved), and green (achieved).

Eligible participants in the BiBBS cohort will be individually randomised 1:1 to the intervention or control arms, with stratification by child age (1 or 2 years old at the time of randomisation) and ethnicity (White British, South Asian, or other). BiBBS researchers will seek consent from participants randomised to the intervention to pass their contact details onto Incredible Years’ delivery agents.

**Discussion:**

This feasibility study will inform the utility of the TwiCs approach within an experimental birth cohort to evaluate interventions for infants, toddlers, and their families.

**Trial registration:**

The study was prospectively registered on ISRCTN (ISRCTN16150114).

**Supplementary Information:**

The online version contains supplementary material available at 10.1186/s40814-023-01441-9.

## Background

### Importance of evaluating early years parenting programmes

Experiences in the first 1000 days of life are important influences on a child’s development, with disruptive experiences within this period being a significant risk for later developmental difficulties [1, 2]. Of particular importance are early attachment and emotional care, which are key for the development of later social and emotional wellbeing and child mental health [3]. Early intervention through parenting programmes has the potential to improve the development and wellbeing of children through parent education [2]. Whilst there are some group-based parenting programmes that evidence reductions in child conduct problems and improvements in child socio-emotional wellbeing for children aged 3 years or older [4, 5], evidence remains weak for parenting programmes for children under 3 years old. Evidence concerning effectiveness in the longer term and in specific subgroups of parents is also weak [2, 4, 6].

Randomised control trials (RCTs) are considered the ‘gold standard’ for establishing intervention effectiveness [7]. However, implementing them in the context of evaluating early years parenting programmes can pose several challenges. First, even if a parent is allocated to the control group of a trial, it may not be possible to control their access to an intervention if they are aware of its existence (e.g. via the Internet, or through books) [8]. Second, ‘disappointment bias’, or the ‘Hawthorne effect’, is sometimes observed when participants are randomised to the control, as opposed to the intervention arm, possibly impacting how they respond to trial measures. Third, they can suffer from a lack of generalisability, as participants who volunteer to participate in RCTs are generally not representative of the wider population that might be eligible to receive the intervention, although the practical importance of this varies considerably across settings [7, 8]. Finally, RCTs are susceptible to a high number of participants being lost to follow-up [7], especially when a study aims to explore long-term effects of the intervention.

### The Trial Within a Cohort Study (TwiCS) design

An alternative design developed with the intention of overcoming some of these issues is a Trial Within a Cohort Study (TwiCS) design (also known as cohort multiple randomised control trial (cmRCT)), where a randomised trial is nested within a cohort study. A TwiCS uses a large observational cohort where cohort participants have consented to be randomly allocated to interventions. This allows for a proportion of this eligible population to be selected at random to be offered the intervention of interest [9].

The TwiCS design adopts a staged consent process, where participants provide initial consent at cohort enrolment that includes an agreement to undergo random selection for future trials and for use of their data as control data in the event of non-selection, without further notification. Participants selected for the intervention are then asked to provide consent at a second stage, specifically related to the trial intervention [10]. This removes the possibility of ‘disappointment bias’ or the Hawthorne effect, as participants in the control arm have already consented to control status and are not aware that they are in a trial. It also reduces the possibility of ‘contamination’, i.e. a parent enrolling in a programme when they are in the control arm, since they will not have received information about the intervention. A further advantage of staged consent is that it provides participants the opportunity to consent at each stage of the study, whilst in conventional RCTs consent for both aspects is asked at the same time. Unlike an RCT, this gives greater insight into which participants refuse consent to being in a cohort (stage 1), and which participants only refuse a particular intervention (stage 2) [10]. An additional benefit of this approach is that this consent process is person-centred and aims to replicate real-world practice, where only those who have access to the intervention being trialled are given information about the intervention.

The presence of an existing cohort means the TwiCS design can implement stratified sampling methods more readily than an RCT utilising conventional recruitment pathways. This can in principle be used to obtain a more representative sample, potentially aiding generalisability. A conventional RCT can only recruit those who are willing to be allocated to either arm [11], whereas a TwiCS can include all participants who are willing to enrol in the cohort, and it appears that the staged consent process may lead to higher recruitment rates [12]. Finally, a cohort consents to access routinely collected data. This means that long-term outcomes will generally be available from routinely collected sources, which reduces the participant burden of extra data collection, and potentially reduces attrition. Several feasibility and full-scale TwiCS have been completed in healthcare settings, including cancer treatments and Huntington’s disease [13–15]. However, the method is yet to be tested within the context of early years interventions.

### Current study rationale

This protocol outlines our plans to test the feasibility of a TwiCS design to offer a group-based parenting intervention to participants enrolled in an interventional birth cohort. The cohort is the Born in Bradford’s Better Start (BiBBS) cohort, an interventional birth cohort recruiting expectant parents in three areas in Bradford [16–18].

The intervention is the Incredible Years Toddler (IY-T) programme, a group-based parent education programme for parents of children aged 1–3 years [19]. RCTs of IY parent programmes for older children demonstrate evidence of positive short-term outcomes [6, 20], whilst the more recently developed IY-T has less evidence in comparison. There are only two previous studies which have investigated the effectiveness of IY-T [21, 22]; however, these studies did not represent socioeconomically and ethnically diverse populations and did not examine any outcomes beyond 12 months. More evidence is therefore needed on the long-term effectiveness of IY-T, particularly in socioeconomically and ethnically diverse populations.

A feasibility study is necessary at this stage due to four key uncertainties about the TwiCS design in this setting. First, IY-T delivery agents usually receive their referrals from their work with the local community. Implementing a TwiCS will introduce a procedural change for both the BiBBS team, and for IY-T’s normal referral processes. Second, due to the timing of recruitment to BiBBS and the timing of recruitment to the intervention (where participants are eligible when they have a child aged 12–36 months old), participants will be contacted between 12 and 36 months *after* they have enrolled in the BiBBS cohort. This delay introduces an uncertainty about the rate of intervention take up. Regular NHS tracing means that participants’ home addresses and children’s health status will be up to date, but phone numbers may have changed. Third, contamination (where participants who are allocated to the control group receive the intervention) may occur in this study since IY-T will still receive their usual referrals from the community during the implementation of the TwiCS; however, the level at which this may occur remains unknown. Finally, previous RCTs of parenting programmes have suffered poor take up and attendance of the intervention, particularly for parents in disadvantaged areas, and parents with a higher level of need (e.g. due to low mood) [23, 24]. We therefore need to test the rates of participation and completion of the intervention to inform the feasibility of a larger evaluation, particularly in a disadvantaged setting.

In line with previous feasibility TwiCS studies, we will test the feasibility of recruiting to the trial, the acceptability of trial processes, and the acceptance rate of the intervention and resulting sample sizes. It is not necessary for us to test the feasibility of recruiting to a cohort, nor the fidelity of intervention delivery [25–27], since the BiBBS cohort study already successfully recruits a large, representative, and diverse sample of mothers [17], and IY-T has been delivered with high fidelity in the study site since September 2018.

### Objectives

This study aims to establish whether it is feasible to conduct a TwiCs evaluation of a parenting programme for parents of toddlers initially recruited during pregnancy into the BiBBS birth cohort.

The specific objectives address key uncertainties. A Red, Amber, Green (RAG) rating system has been applied to support the feasibility assessment of each objective: to be rated red (not achieved), amber (partly achieved), and green (achieved). The objectives are:

1. To establish whether TwiCS methodology can be implemented to create a control and intervention arms, whilst documenting any incidences of contamination (i.e. control participants that are offered or receive the intervention).
2. To establish whether satisfactory rates of intervention uptake are achieved among participants allocated to the intervention.
3. To establish whether satisfactory rates of retention of participants in the intervention can be achieved.

## Methods

### Design

We have used the SPIRIT checklist for producing this protocol and made amendments based on the CONSORT ROUTINE guidelines for cohort RCTs, and the guide to reporting protocols for pilot and feasibility studies [28–30].

This study aims to assess the feasibility of a TwiCS evaluation of the IY-T programme using BiBBS cohort participants. The TwiCS will have two arms with a 1:1 random allocation ratio (intervention to control). Although this deviates from the ‘random selection’ method proposed by Relton et al. (2010), this approach is still in line with the TwiCS methodology, as randomisation is occurring from a cohort of participants, and consent is staged [31].

### Cohort

The cohort is the Born in Bradford’s Better Start (BiBBS) birth cohort which recruits pregnant women and their newborn babies from the three inner city Better Start Bradford areas. Better Start Bradford is an initiative which provides a range of interventions for expectant families and families with children aged 0–3 in Bowling and Barkerend, Bradford Moor and Little Horton (three areas within Bradford). Most of the Better Start Bradford area falls into the most deprived 10% of areas in England (Bradford Council, 2019; GOV.UK, 2019). Bradford district is the 13th most deprived local authority of 326 in England (City of Bradford Council, 2019, 2021).

Pregnant mothers are eligible for recruitment to BiBBS if they are living in the Better Start Bradford area and are registered to give birth at Bradford Teaching Hospital NHS Foundation Trust (BTHFT) [16]. Upon recruitment, women complete an in-depth baseline questionnaire during pregnancy and consent to routine linkage to both their own and their child’s health and education records and records of their participation in Better Start Bradford interventions. BiBBS recruits 54% of the eligible population and is representative of the eligible pregnant population in terms of ethnicity, age, area deprivation, English language ability, and parity [17]. The intervention and control participants in this study are therefore also considered to be representative of this area [17]. All BiBBS women that are eligible to receive the IY-T programme, and have not yet received it, will be identified and form the eligible population.

### Consent

When recruited into the BiBBS cohort, participants are asked to sign a consent form, which includes permission for researchers to access their routine data and to be randomly selected to receive an intervention or to act as a control to evaluate that intervention, in line with guidance on TwiCS designs [9]. The consent statement was developed with the Community Research Advisory Group to ensure clarity and understanding with participants and states: “I understand that if there are not enough places for everyone to take part in Better Start Bradford projects, families may be selected to take part by chance (randomly). If my family are not selected to take part in a project, information on me and my child may be compared with families who have been selected to take part” (see Consent form for Pregnant Women version 4, 21.02.18).

**Fig. 1:**
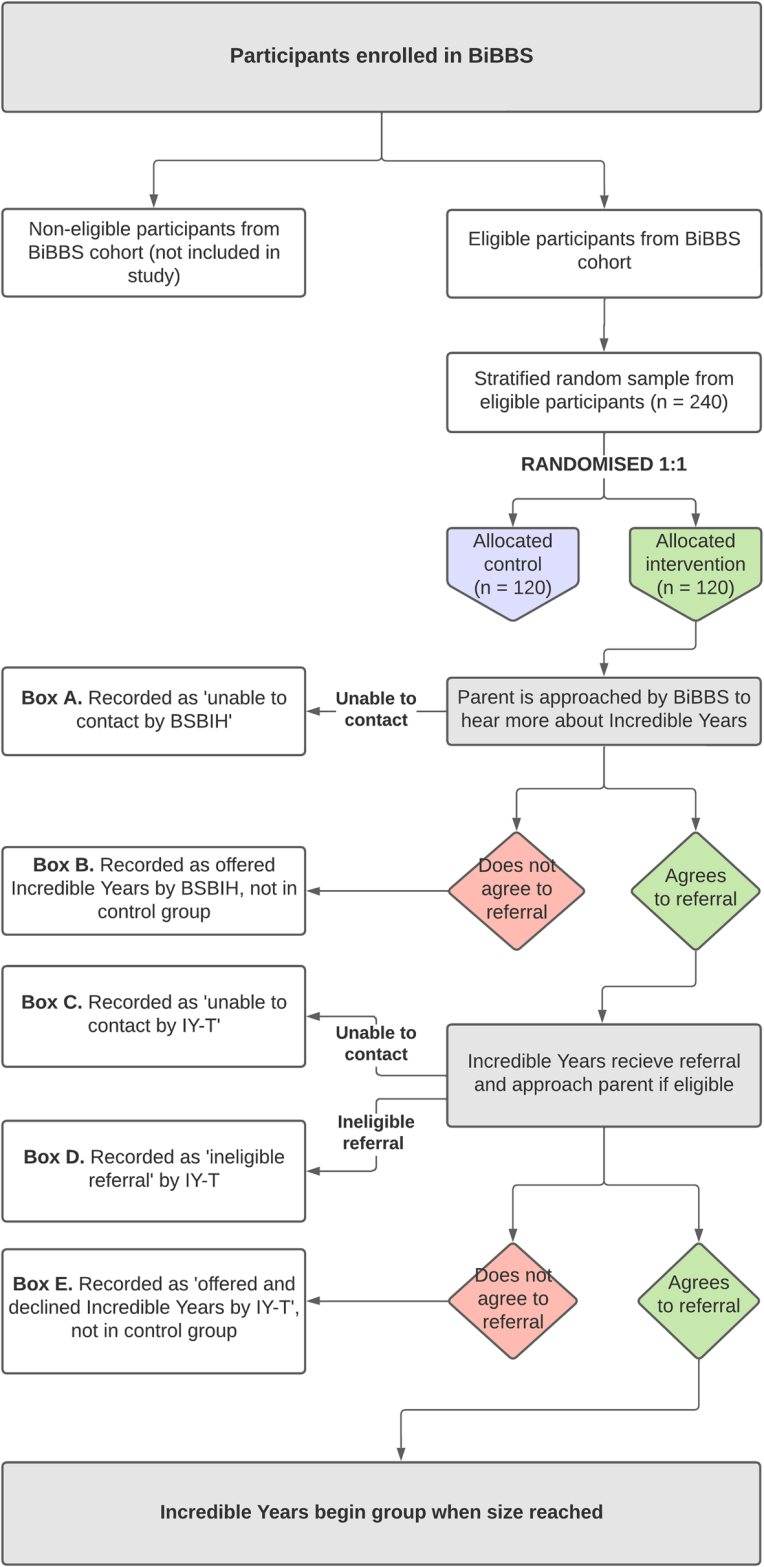
Flow chart describing the implementation of the feasibility TwiCS procedures

In line with TwiCs design, additional consent is not required for the random selection of participants from the BiBBS cohort for this study (see Fig. 1) [9]. BiBBS participants are informed that they have the right to decline consent without having to provide a reason, and are able to withdraw from the study at any time. This study will follow the BiBBS processes of study withdrawal [16]. Any participants who withdraw from BiBBS prior to the TwiCS processes will not be included in this study. If participants withdraw consent after they have been randomised into the IY-T TwiCS, their outcome data will be included in the final study analysis unless they specifically request that this data not be used.

### Research ethics approval

The protocol for BiBBS recruitment and collection of routine outcome data was approved by Bradford Leeds NHS Research Ethics Committee (15/YH/0455). Research governance approval was gained from Bradford Teaching Hospitals NHS Foundation Trust. The existing ethics includes approval for the evaluation of Better Start Bradford, including the use of cohort participants to create control groups. It also states that full details of any TwiCS or RCTs will be submitted as sub-study amendments to the existing protocol. The protocol for the current sub-study has been approved by the Bradford Leeds NHS Research Ethics Committee as an amendment to the existing BiBBS protocol.

### Intervention

Better Start Bradford commissioned Barnardo’s (a large UK-based children’s charity) to provide and deliver Incredible Years Toddler (IY-T) in the study site (https://www.barnardos.org.uk/). IY-T covers 8 key topics such as ‘social and emotional coaching’ and ‘the art of praise’ which are delivered through 2-h sessions over 13 weeks by two trained group facilitators who promote peer support and shared learning. The programme ultimately aims to promote parent-child interactions and positive parenting strategies for participating parents. This is thought to promote improved social and emotional development and support children to enter school with improved language and communication skills. Prior to the programme starting, parents receive 3 promotional contacts, consisting of telephone contact and at least 1 home visit. The initial telephone contact will introduce the parents to the project and facilitators and build their confidence in attending. The home visit builds rapport between the family and facilitators and involves the completion of pre-course questionnaires and identification of any barriers that families might have in accessing the group such as crèche, language difficulties or concerns about what the group might involve. For more detail on the theory of change, content and delivery of the Incredible Years Toddler intervention, please see the programme manuals (https://incredibleyears.com/books/incredible-toddlers/).

Group facilitators attend an initial 3-day training in the programme, and guidance from the programme developed recommends that facilitators should also engage in regular supervision and pursue official accreditation. The accreditation process is rigorous, requiring group facilitators to provide evidence of delivery including video footage of sessions and various forms and checklists. The delivery agents currently have six facilitators in total, two of which are accredited, and two of which are currently in the accreditation process. The programme is delivered in a combination of face-to-face and virtual formats (dependent on lockdown rules in place at the time of the study and participant needs). Barnardo’s have delivered IY-T with high levels of intervention fidelity and have engaged in regular supervision and successfully achieved accreditation for two of their group facilitators [32]. We have been conducting monitoring and evaluation of the programme for several years (reports available at https://bsbinnovationhub.wordpress.com/incredible-years/).

### Trial participants

We use the term ‘women’ to refer to birthing parents; we recognise, however, that not all birthing parents are women. For this feasibility TwiCs of IY-T, women are eligible if they:

- Provided consent to the BiBBS cohort study and agreed to be contacted for future research
- Have not withdrawn consent to the BiBBS cohort at the time of randomisation
- Are still living in the Better Start Bradford area at the time of randomisation
- Have one or more BIBBS child(ren) aged between 12 and 36 months at the time of randomisation
- NHS tracing confirms that their child is still living with them, and is alive
- have not already received IY-T in the Better Start Bradford area for any of their children^1^

### Implementation

Figure 1 presents how study participants will be drawn and randomised from the BiBBS cohort. A Standard Operating Procedure (SOP) for recruitment of participants was developed for this study. Participants randomised to intervention will initially be contacted by a designated researcher from the BiBBS team, who will confirm their eligibility and ask if they consent to their details being passed onto the IY-T team at Barnardo’s. Those who cannot be contacted after four attempted contacts will be recorded as ‘unable to contact’ (Box A). For each individual woman who is contacted, verbal consent will be sought for their details to be shared with the Incredible Years team. Those who are contacted, but who decline consent will not be followed up and will be recorded as having been offered the intervention but declined (Box B).

For those who agree to the referral, the BiBBS team will pass on the referral to the IY-T team who will then re-contact them by telephone. If participants are not able to be contacted at this stage, they are recorded as such (Box C). If IY-T assesses the referral as ineligible, they are recorded as such (Box D); this should be unlikely, however, circumstances may change, e.g. a child becomes ‘too old’ or a child is removed from the parents. We will therefore record any occurrences of this, with reasons where possible. If they can be contacted, this phone call will allow the IY-T team to provide them with more detail about the specific programme available to them and check the eligibility of these participants. If the participant accepts the offer, a home visit with one of the IY facilitators will be arranged. More information about the programme will also be sent out to all women who accept the offer of a home visit. If parents initially accept the offer from BiBBS, but not from IY-T, they will be recorded as having been offered the intervention but declined (Box E).

The numbers randomised (*n* = 240) and offered IY-T (*n* = 120) in this feasibility study aim to fill two IY-T courses (*n* = 24). If the number of study participants is not sufficient to support optimal group sizes (12 per group), the IY-T team may ‘top up’ groups by including parents referred to them from other services. These parents are not study participants and will not be included in the research.

### Patient and public involvement

Members of the Bradford Community and Research Advisory Group (CRAG) advised on the consent statement and study design for recruitment to the BiBBS cohort, and any processes regarding recruitment and implementation of this TwiCS involved the IY-T delivery team. The CRAG are also involved in the interpretation and dissemination of all BiBBS findings, and they will be for results relating to this study.

### Randomisation

Eligible participants in the BiBBS cohort will be individually randomised 1:1 to the intervention (*n* = 120) or control group (*n* = 120), using blocked randomisation with stratification by child age (1 year or 2 years at the time of randomisation) and ethnicity (White British, South Asian, or other). The six allocation sequences (one for each stratum) will be generated using Stata/SE v17.0 (for Windows 64-bit x86-64) or later, using the user-written Stata command ralloc (Ryan, 1997).

We will draw a stratified random sample of 240 participants from all eligible participants in the BiBBS database, with sampling fractions (approximately) proportional to the distribution of the six randomisation strata in the eligible cohort population. The *n*_*i*_ sampled participants in stratum *i* will then be randomly sorted and matched against the first *n*_*i*_ allocations in the allocation sequence for stratum *i*.

### Statistical methods

The feasibility study will apply the CONSORT-ROUTINE reporting guidelines and the flow diagram where relevant. As this is a feasibility study, reporting guidelines about the intervention outcome are not relevant and will not be described for this study. A future trial would use data that can be routinely linked and accessed as an outcome.

The feasibility outcomes are described in Table 2. These will be reported descriptively using raw numbers and percentages, with 95% Wilson binomial confidence intervals to provide some indication of the uncertainty associated with the observed point estimates.

We will also report a description of the selected intervention and control participants with regard to their sociodemographic characteristics and compare feasibility outcomes across sociodemographic groups where possible. We will suppress any counts of <5 to protect participants’ identity. All counts of missing data will be reported where known, with reasons where possible.

### Sample size

The IY-T service delivery team have the capacity to deliver two groups for this feasibility TwiCS. Each IY-T group aims to recruit 12 parent-child dyads (hereafter referred to as enrolees) and retain 10 participants. We have therefore based our sample size to be randomised (*n* = 240) on achieving these numbers (24 enrolees total, or 12 enrolees per group) (see below for details on how these numbers have been derived).

### Feasibility outcomes

We have based the feasibility outcome targets on data from two key sources. The first relates to a postnatal data collection sweep conducted as part of the main BiBBS cohort study. The second is information on rates of IY-T enrolment, participation and completion (where an ‘enrolee’ is referred and seen face to face in at least one pre-course contact, a ‘participant’ is someone who attends at least 1 week of the groups, and a ‘completer’ is someone who attends at least 8 of the 13 group-based sessions) reported by Barnardo’s and the Better Start Bradford Innovation Hub (see https://borninbradford.nhs.uk/what-we-do/improving-health/bsb-innovation-hub/) Table 1.

**Table 1:** Target figures

Projected target figures	Rationale for target figures
120 randomised to intervention → 84 women are contactable]	80% of women were contactable for the BiBBS postnatal sweep, which takes place approximately 12 weeks after recruitment into BiBBS. We will contact women 1–3 years after recruitment into BiBBS, and so we have set our rate to be lower at 70%.
84 women contacted → 44 women consent to the referral → 22 women enrol	On average, the rate of conversion from referral to enrolee in IY-T is 52%. We therefore anticipate that 52% of randomised BiBBs participants will agree to be referred, and 50% of those referred will enrol into the programme.
22 enrolees → 20 enrolees go on to participate	On average, 89% of enrolees to IY-T are converted into participants. We have set a target of 90% of randomised enrolees to be converted into participants.
20 participants → 12 participants go on to complete	60% of participants go on to complete IY-T. We have set our rate to be 60%.

**Table 2:**
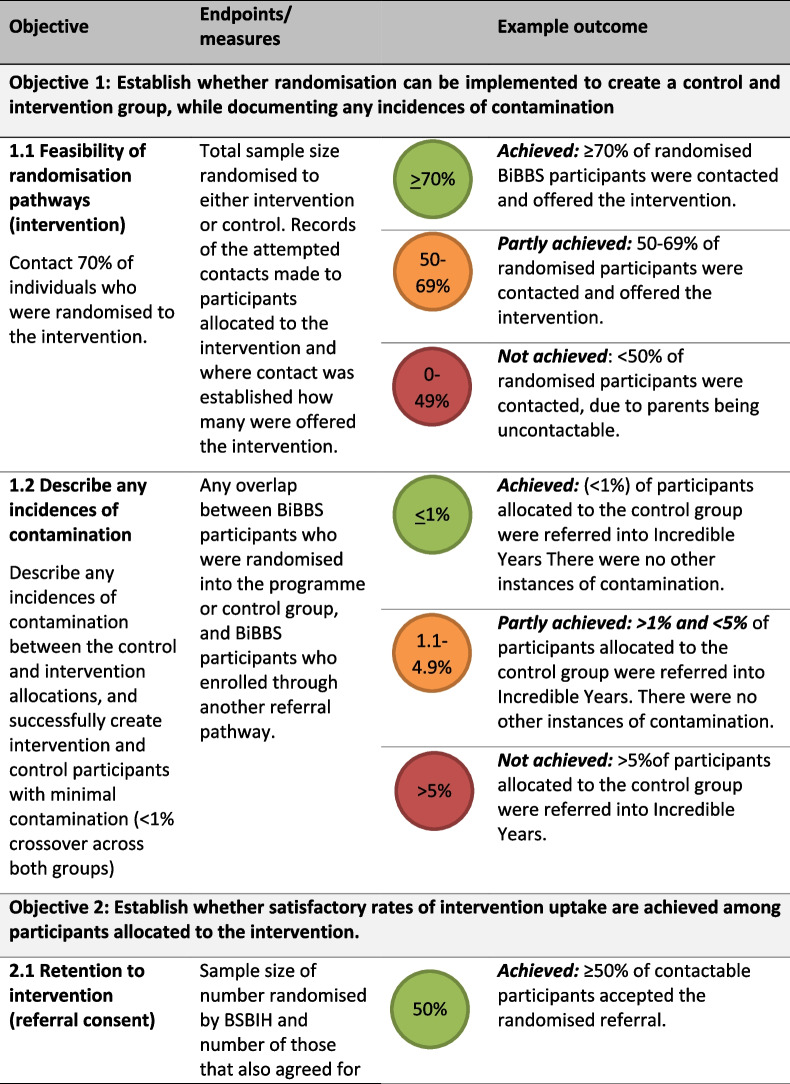

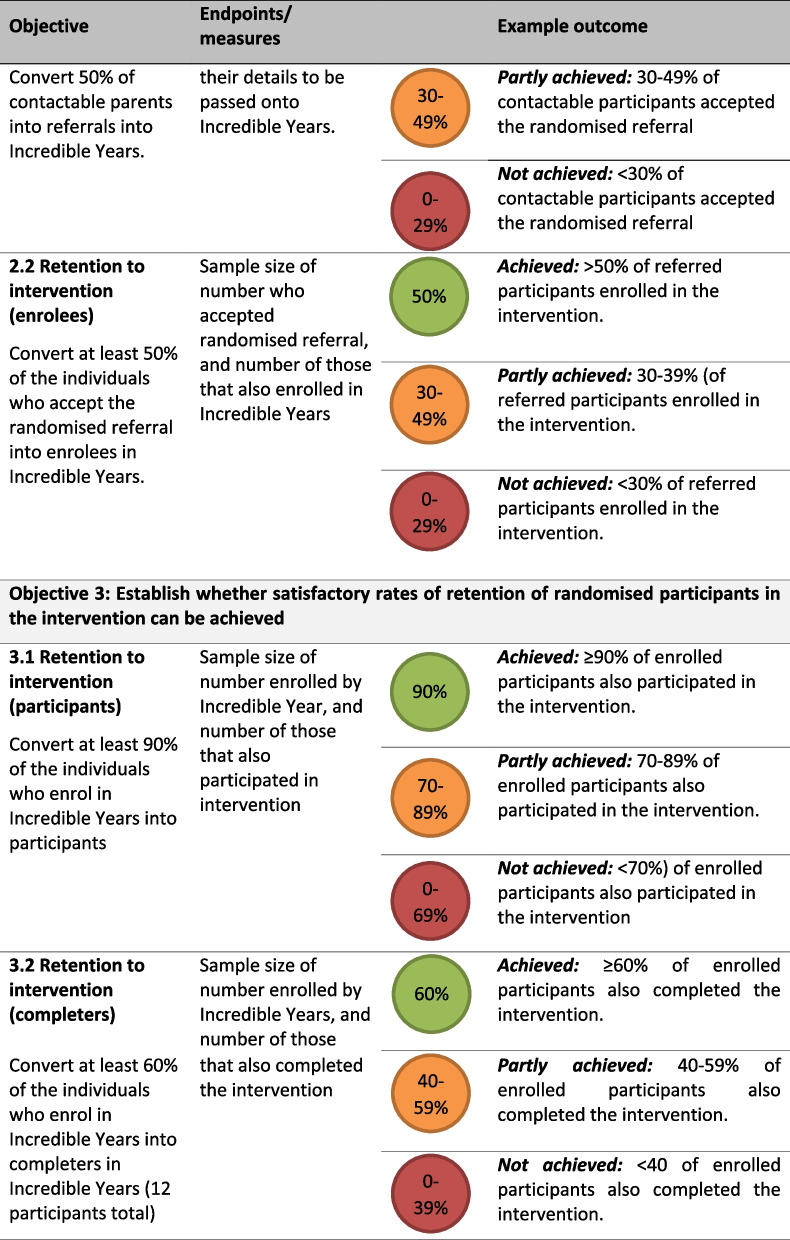
Feasibility objectives and outcomes

Table 1 summarises our target figures and how these were decided upon, and Table 2 summarises these targets and outlines how feasibility objectives will be assessed. In addition to the above, we also aim to assess contamination (any overlap between allocated control group, and non-randomised intervention group participants) and aim for this to occur in <5% of the sample. This is based on an estimate that <5% contamination has a negligible effect on the required sample size for a trial [33]. A RAG rating system has been applied to support the feasibility assessment of each objective: to be rated as green (achieved), amber (partly achieved) and red (not achieved). For an objective to be achieved and rated green, it must reach the percentage level specified above (e.g. 70% for contactable women, 50% for converting randomised participants into referrals). The levels at which we reach amber are equal to the green target, minus 20%. This is except for the rate for contamination (objective 1.2), where the rates are set on achieving less than 5% contamination.

### Trial management

The trial is considered low risk and oversight will be provided by the Incredible Years working group and the programme management group of the Innovation Hub. The study research team will be responsible for the allocation of participants to interventions, routine data linkage, and all data analysis. All other elements of the study will be performed by individuals external to the research team as detailed below.

Recruitment into the BiBBS cohort is currently undertaken by a team of multilingual Community Research Assistants. The BiBBS community research assistants will offer the referral to IY-T for BiBBS participants. This team has Standard Operating Procedures (SOPs) for contacting and enrolling parents into the intervention. Enrolment of participants onto the IY-T programme will be the responsibility of the project co-ordinator for the IY-T delivery team based at Barnardos, using their usual recruitment procedures.

### Data collection and management

The project-level data regarding the number of women who enrol, participate, and complete the programme will be linked to information on BiBBS participants and used in this feasibility TwiCS.

As per the BiBBS protocol, record matches for routine data linkage will be validated on the basis of NHS number plus multiple non-unique identifiers (e.g. surname, date of birth) where possible. The central database, hosted by BTHFT, will store data obtained from Medway and SystmOne routine health and education data. Data from each source will be linked at the BiBBS person level and will be structured and maintained by BiBBS data managers as a long-term strategic store to service cohort data capture and analysis. The entire database schema and data will be backed up nightly. Further details of the routine data management process can be found in the BiBBS protocol.

### Monitoring

#### Harms

There is unlikely to be harm to individuals from taking part in the intervention as it is non-invasive, though is possible that discussions relating to family relationships could highlight potential safeguarding issues for participating parents and/or their children. To mitigate this risk, the practitioners delivering the intervention and community research assistants making the phone calls are trained in safeguarding. The BiBBS study protocol has been approved by the Bradford and Leeds Research Ethics Committee (15/YH/0455). All BiBBS researchers follow Bradford Teaching Hospital Foundation Trust’s Safeguarding Adults and Safeguarding Children policies.

#### Data monitoring and auditing

This study evaluates an intervention that is already being commissioned by BSB and implemented independently from this study and the evaluation team. The Innovation Hub is conducting regular monitoring of the intervention including reporting on progression criteria that have been agreed with the intervention and Better Start Bradford teams. This information is used by Better Start and the intervention team to inform commissioning and implementation decisions. There are also a number of project management groups for BiBBS conducted within BTHFT described in Additional file 1 in the BiBBS protocol [16]. The sponsor of BiBBS is the BTHFT Research Management and Support Office which may conduct independent auditing of the study.

#### Dissemination policy

Findings will be produced in reports and shared among relevant BSB partners and commissioners in Bradford and England. A briefing will be shared with BiBBS participants through existing communication strategies (including website, social media, newsletters, and birthday cards. Summaries of findings may also be widely reported in the local BSB communities using BiBBS and BSB newsletters, social media, and local press. In addition, the findings from this study will be submitted for publication in scientific journals and as conference abstracts.

## Discussion

Parenting programmes delivered in the early years have the potential to reduce the onset of child mental health difficulties [1, 2]. It is therefore crucial that we find efficient, implementable methods to establish the effectiveness of such programmes. A TwiCS design would overcome many of the limitations of the traditional RCT design and has the advantage of a staged consent process and access to long-term routine data to use as outcomes [9, 10].

The BiBBS cohort presents a unique opportunity to test the feasibility of a TwiCS within an ethnically diverse and deprived community. Given the difficulties of recruiting more disadvantaged populations to trials [23], this feasibility study will give useful information about whether a TwiCS is feasible with such populations. If it is found to be feasible, a larger TwiCS with a sample sufficiently large enough to enable precise estimation of intervention effectiveness could be designed and implemented. A future TwiCS would have the advantage of using routinely linked healthcare and educational data as an outcome, such as the Ages and Stages Questionnaire (ASQ) collected during mandatory 2–2 ½ year health visitor visits [34], or the Early Years Foundation Stage Profile (EYFSP) reported by teachers at the end of a child’s reception year [35]. A fully powered TwiCS evaluation of the IY-T parenting programme would have the potential to improve the evidence base of parenting programmes for parents of children under 3 years old [2, 4, 6].

Overall, the findings from this feasibility TwiCS will be useful for future studies that wish to apply a TwiCS design to ascertain the effectiveness of interventions delivered in the early years, both within and beyond the BiBBS cohort.

### Supplementary Information

**Additional file 1.** SPIRIT statement/checklist with amendments from CONSORT pilot trail and CONSORT ROUTINE statements. Description: Word document containing the SPIRIT statement with amendments from CONORT pilot trial and CONSORT ROUTINE statements.

## Data Availability

These data cannot be shared publicly as they are available through a system of managed open access. Researchers are encouraged to make use of the BiBBS data, which are available through a system of managed open access. Before you contact us, please make sure you have read our Guidance for Collaborators. Our BiB Executive reviews proposals monthly and we will endeavour to respond to your request as soon as possible. You can find out about the different datasets in our Data Dictionary. If you are unsure if we have the data that you need, please contact a member of the BiB team (borninbradford@bthft.nhs.uk). Once you have formulated your request please complete the ‘Expression of Interest’ form available here and send to borninbradford@bthft.nhs.uk. If your request is approved, we will ask you to sign a Data Sharing Contract and a Data Sharing Agreement, and if your request involves biological samples, we will ask you to complete a material transfer agreement.

## Abbreviations

BiBBS: Born in Bradford’s Better Start
IY-T: Incredible Years Toddler
TwiCS: Trial within a Cohort Study
RCT: Randomised control trial
BSB: Better Start Bradford

## Footnotes

1 The index child is the child that is documented to be taking part in IY with their parent. The parent chooses which child they wish to record as their index child. This is relevant for parents who have >1 BiBBS child.
